# Are there any indications of transoral odontoidectomy today?

**DOI:** 10.3171/2020.4.FocusVid.20332

**Published:** 2020-07-01

**Authors:** P. Sarat Chandra

**Affiliations:** Department of Neurosurgery, All India Institute of Medical Sciences (AIIMS), New Delhi, India

The use of transoral odontoidectomy has been reduced significantly following the introduction of techniques such as direct posterior intraoperative reduction, realignment, and fixation.^1–7,9,12,13^ It may, however, be performed in cases when posterior-only procedures are not useful enough to reduce the spinal compression completely. In cases where C1 is not occipitalized, a C1–C2 screw fixation is usually enough to reduce the atlantoaxial dislocation. Congenital cases where the C1 is fused with the occiput are associated with moderate to severe basilar invagination and atlantoaxial dislocation.

In fact, performing a transoral odontoidectomy may be an elective option, following partial reduction after a posterior procedure. This allows for an easier transoral procedure. In the current scenario, the following may be indications of transoral procedures: 1) An anterior release followed by posterior fixation: Wang et al.^15,20^ and some others have suggested this procedure. They felt that an anterior release of ligaments would allow a better posterior reduction and realignment. However, in most cases this is not required and a direct reduction and fixation provides excellent alignment. 2) An incomplete or nonreduction following a posterior approach: this is currently the most common indication for a transoral approach. 3) Reduction and fixation from the anterior approach: Some authors have suggested an anterior-only approach to reduce and perform fixation from C1–2 from the anterior route.^14^

Some important things to consider prior to transoral odontoidectomy include the following: 1) Consider the degree of mouth opening. This should be about three fingers breadth to allow ingress to the surgical approach. 2) Consider examining the oral cavity; exclude any infective conditions and assess oral hygiene prior to surgery. These aspects may be missed when considering surgery.

Neuronavigation has emerged as a useful optimal surgical aid while performing surgery.^16–19^ This is important especially to delineate the inferior part of the C2 body, as has been shown in this video. The other uses of neuronavigation are to determine the lateral extent. Often, if the surgeon is not experienced, the lateral portion of the base of dens is left behind, and this may cause postoperative compression.

The endoscopic transoral procedure^8,10,11,21^ is also emerging as a useful alternative to perform an odontoidectomy. The advantage, of course, is that it is minimally invasive and not dependent on the degree of mouth opening.

In summary, the role of transoral odontoidectomy is shrinking fast, but there are still some indications for it, as mentioned.
